# Single-cell and spatial transcriptomic profiling of cardiac fibroblasts following myocardial infarction

**DOI:** 10.1038/s41597-025-06533-0

**Published:** 2026-01-13

**Authors:** Silvia C. Hernández, Marina Ainciburu, Laura Sudupe, Nuria Planell, María López-Moreno, Amaia Vilas-Zornoza, Luis Diaz-Martinez, Jorge Cobos-Figueroa, Juan P. Romero, Sarai Sarvide, Patxi San Martin-Uriz, Ana López-Pérez, Gloria Abizanda, Purificación Ripalda-Cemboráin, Emma Muinos-López, Vincenzo Lagani, Jesper Tegner, Ming Wu, Stefan Janssens, José M. Pérez-Pomares, Felipe Prósper, Adrián Ruiz-Villalba, David Gómez-Cabrero

**Affiliations:** 1 The Wilf Family Cardiovascular Research Institute, Department of Medicine (Cardiology), Department of Microbiology and Immunology, Albert Einstein College of Medicine, Bronx, NY USA; 2 Hemato-Oncology Program, Cima Universidad de Navarra, Cancer Center Clínica Universidad de Navarra (CCUN), Instituto de Investigación Sanitaria de Navarra (IdiSNA), Pamplona, Spain; 3 Bioscience Program, Biological and Environmental Sciences and Engineering Division (BESE), King Abdullah University of Science and Technology (KAUST), Thuwal, Saudi Arabia; 4 Computational Biology Program, Cima Universidad de Navarra, IdiSNA, Pamplona, Spain; 5 Department of Animal Biology, Universidad de Málaga, Málaga, Spain; 6 Instituto de Investigación Biomédica de Málaga (IBIMA-Plataforma BIONAND), Málaga, Spain; 7 Centro de Investigación Biomedica en Red de Cancer (CIBERONC), Madrid, Spain; 8 Centro de Supercomputación y Bioinnovación (SCBI), Universidad de Málaga, Málaga, Spain; 9 Navarrabiomed, Fundacion Miguel Servet, Universidad Pública de Navarra (UPNA), IdiSNA, Pamplona, Spain; 10 Regenerative Medicine Program, Cima Universidad de Navarra, IdiSNA, Pamplona, Spain; 11 Department of Orthopedics, Clinica Universidad de Navarra, IdiSNA, Pamplona, Spain; 12 SDAIA-KAUST Center of Excellence in Data Science and Artificial Intelligence, Thuwal, Saudi Arabia; 13 Institute of Chemical Biology, Ilia State University, Tbilisi, Georgia; 14 Unit of Computational Medicine, Department of Medicine, Center for Molecular Medicine, Karolinska Institutet, Karolinska University Hospital, L8:05, SE-171 76, Stockholm, Sweden; 15 Computer, Electrical and Mathematical Sciences and Engineering Division, KAUST, Thuwal, 23955-6900 Saudi Arabia; 16 Science for Life Laboratory, Tomtebodavagen 23A, SE-17165 Solna, Sweden; 17 Department of Cardiovascular Sciences, Clinical Cardiology, KU Leuven, Leuven, Belgium; 18 Hematology and Cell Therapy Department, Clinica Universidad de Navarra, CCUN, IdiSNA, Pamplona, Spain

**Keywords:** Mechanisms of disease, Heart failure, Experimental models of disease, High-throughput screening, Translational research

## Abstract

Cardiac fibroblasts (CFs) are key mediators of heart repair following myocardial infarction (MI). A specific CF subpopulation, termed Reparative Cardiac Fibroblasts (RCFs), has been shown to orchestrate scar formation and prevent ventricular rupture after MI. However, the timing of RCF appearance and the molecular events underlying this transition remain largely undefined. Here, we present a multi-modal dataset capturing the transcriptional dynamics of CFs during the early phase post-MI. Our integrative dataset combines bulk RNA sequencing, RNAscope *in situ* hybridization, and spatial transcriptomics to anatomically and temporally map the gene expression changes associated with the transition into RCFs. The dataset provides resources to characterize the distinct molecular programs that guide the emergence of RCFs from Periostin (*Postn*)^+^ activated CFs. This dataset provides a valuable resource for investigating CF heterogeneity and reparative pathways following MI. All raw and processed data, along with detailed metadata and annotations, are made available to facilitate reuse by the cardiovascular and single-cell biology communities.

## Background & Summary

Fibrosis can be considered as an evolutionarily conserved adaptative tissue response to damage^1^. This phenomenon is characterized by a deep remodeling of the extracellular matrix (ECM) architecture and is led by fibroblasts. Although fibroblasts have been historically considered as a homogeneous population, recent studies have demonstrated their heterogeneity^2–9^. Understanding this heterogeneity is key to unraveling the cellular and molecular mechanisms that drive the fibrotic process and thus modulate it. In the heart, the progression of the fibrotic tissue, increasing ECM deposition, and its transformation into a pathological scar, with the characteristic reduction of the cardiac function and the consequent heart failure, occurs within the first week after MI^10^. Therefore, understanding the cellular and molecular mechanisms that govern early fibroblast responses is critical to enable novel treatments to control this reparative process^11^.

As initial effort to clarify those mechanisms, we recently described a subpopulation of activated CFs (Collagen Triple Helix Repeat Containing-1 (*Cthrc1*)^+^ *Reparative Cardiac Fibroblasts*, RCFs) as the primary driver of the healing process in the context of MI^6^. In that study, we also hypothesized that *Cthrc1*^+^ RCFs represent a final transitional stage of activated *Postn*^+^ CFs. Interestingly, recent results in early phases of the fibrotic process in different organs, including lung and liver, revealed a subpopulation of *Cthrc1*^+^ fibroblasts with a similar transcriptomic profile and also characterized by enhanced ECM production^3,12–14^. Determining whether fibroblast phenotypes and transitional states are organ-specific or represent a conserved response across tissues could inform broader therapeutic strategies^15^.

Despite these parallels, in the heart, the timing, location, and mechanisms underlying CF activation and their posterior transition into RCFs remain unclear. To address those three questions new analysis are required, and to support the study of the post-MI heart, we generated a multi-omics data set —including bulk, single-cell, and spatial transcriptomic profiling— to enable researchers to dissect the transcriptional trajectory into RCFs after MI. Additionally, we validated the utility of the dataset both technically, using RNAscope, and biologically, in independent systems such as porcine models and patient samples, thereby underscoring its translational relevance.

## Methods

### Defining the window of activation of *Cthrc1*^+^ Reparative Cardiac Fibroblasts

**Fig. 1:**
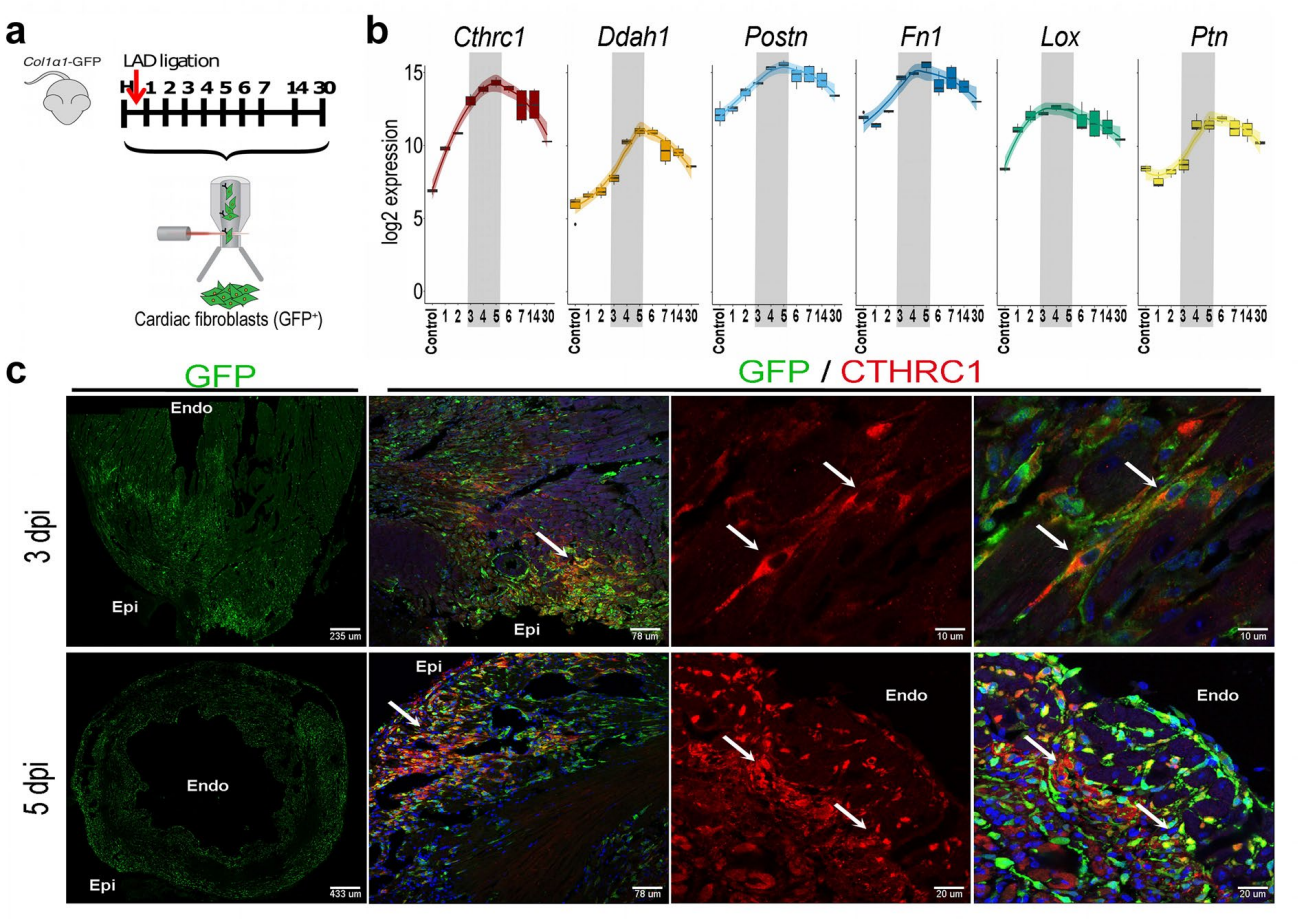
Bulk transcriptomic profiling of cardiac fibroblasts during myocardial infarction. (**a**) Experimental design. Myocardial infarction (MI) was induced in 8–10-week-old *Collagen1α1-GFP* mice (*Col1α1*-GFP)^17^ by ligation of the left anterior descending (LAD) coronary artery. *Col1α1*-GFP^+^/CD31^−^/CD45^−^ cardiac fibroblasts (CFs) were sorted from infarcted hearts at 1–6 days post-infarction (dpi). Bulk RNA sequencing was performed and integrated with previously published datasets^6^ from healthy and infarcted hearts at 7, 14, and 30 dpi. (**b**) Box plots showing gene expression dynamics of the Reparative Cardiac Fibroblast (RCF) gene signature. Grey shading indicates the peak activation period between 3 and 5 dpi. Number of samples for each time point: Control, n = 4; 1 dpi, n = 3; 2 dpi, n = 3; 3 dpi, n = 4; 4 dpi, n = 3; 5 dpi, n = 3; 6 dpi, n = 3; 7 dpi, n = 4; 14 dpi, n = 4; 30 dpi, n = 2. (**c**) Representative immunohistochemistry of CTHRC1^+^ (red) RCFs in the infarct zone of *Col1α1*-*GFP* hearts at 3 and 5 dpi. GFP^+^ CFs are green; nuclei (DAPI) are blue; colocalizations appear yellow. Arrows indicate RCF-like cells. Epi, epicardium; Endo, endocardium.

To generate a dataset enabling the dissection of the activation dynamics of cardiac fibroblasts (CFs) following myocardial infarction (MI), we first required identifying the time windows in which Reparative Cardiac Fibroblasts (RCFs) appears after MI. To this end, we performed novel bulk RNA-seq profiling (and associated bioinformatic analysis) of isolated *Col1α1*- GFP^+^/CD31^−^/CD45^−^ CFs between 1- and 6-days post-infarction (dpi) (Fig. 1a). Additional bulk RNA-seq datasets from the GSE132146 SuperSeries^16^ (healthy, 7, 14 and 30 dpi CF samples) were included in our study^6^. The expression of highly relevant RCF marker genes, such as *Cthrc1*, *Ddah1*, *Postn*, *Fn1*, *Lox* and *Ptn*, peaked between 3 and 5 dpi (Fig. 1b). Accordingly, a few CTHRC1 + CFs were identified in the infarcted hearts at 3 dpi, although an expansion of this subpopulation was observed in both the border and the infarcted zones at 5 dpi (Fig. 1c).

### Single-cell profiling of activation of *Cthrc1*^+^ Reparative Cardiac Fibroblasts

**Fig. 2:**
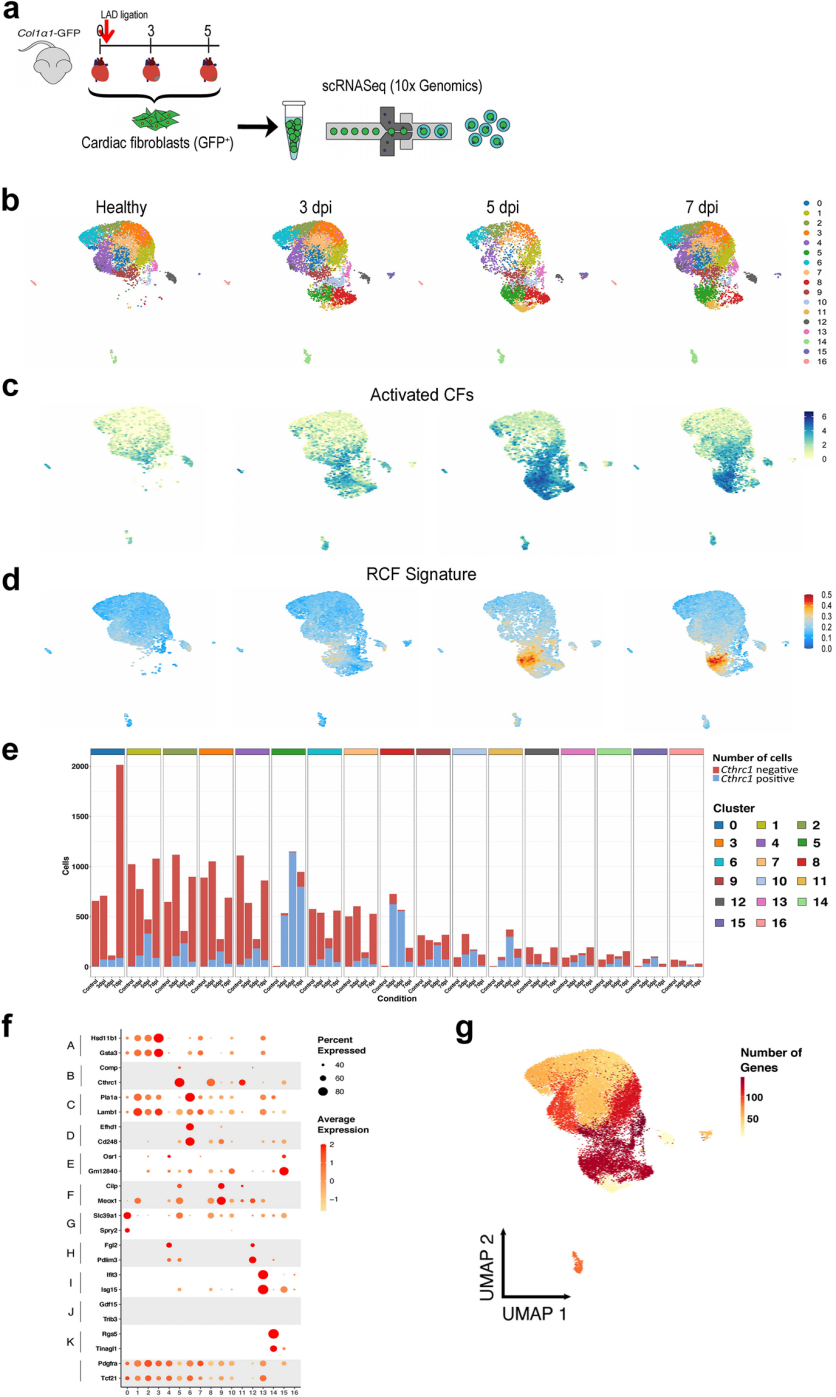
Single-cell transcriptomic characterization of CFs during early post-infarction remodeling. (**a**) Experimental design. *Col1α1*-GFP^+^/CD31^−^/CD45^−^ CFs were sorted from infarcted mouse hearts at 3 and 5 dpi. Droplet-based single-cell RNA sequencing (scRNA-seq) was performed using 10x Genomics technology and integrated with published data^6^ from healthy and 7 dpi infarcted hearts. (**b**) UMAP (Uniform Manifold Approximation and Projection) plots of CF clusters from scRNA-seq across healthy (6,277 cells), 3 (7,888), 5 (4,839), and 7 dpi (8,991) conditions (n = 2 mice per time point). Seventeenth clusters (named as 0–16) were defined by unsupervised analysis. (**c**) UMAP plots showing the distribution of *Postn*^+^ cells (activated CFs) across conditions. (**d**) UMAP plots showing enrichment scores for the RCF genes signature^6^ in individual conditions. Blue represents low enrichment; red indicates high enrichment. (**e**) Bar plots showing the number of *Cthrc1*-negative (red) and *Cthrc1*-positive (blue) CFs in each subpopulation across time points following MI. (**f**) Dot plots showing a comparison between previously defined clusters A-K^6^ (healthy, 7, 14, and 30 dpi) and the newly established ones, 0–16, at healthy, 3, 5, and 7 dpi, based on the expression levels and specificity of two top marker genes/cluster. Dot size indicates the proportion of expressing cells; color intensity reflects normalized gene expression. (**g**) UMAP plot indicating the number of differentially expressed genes from the RCF signature when comparing *Cthrc1*^+^ vs *Cthrc1*^*−*^ cells in each cluster. Color scale represents the number of differentially expressed genes.

Based on these findings, we defined the transition window into RCFs as spanning from 3 to 5 dpi. Next, to generate a high-resolution dataset capturing fibroblast differentiation dynamics, we performed both single-cell and spatial transcriptomic profiling on samples from control (healthy), 3 dpi, and 5 dpi time points. First, we conducted single-cell RNA sequencing (scRNA-seq) on *Col1α1*-GFP^+^/CD31^−^/CD45^−^ CFs isolated at 3 and 5 dpi (Fig. 2a). This newly generated dataset was integrated with previously published scRNA-seq data from our group^6^ (GSE132146^16^), which includes CFs from healthy and 7 dpi hearts. Second, we performed Spatial Transcriptomics using the 10x Genomics Visium platform on heart sections from healthy, 3 dpi, and 5 dpi samples, providing spatial context to the transcriptional changes observed during the transition into RCFs.

### Animal models

All animal requisitions, housing, treatments and procedures were performed according to all state and institutional laws, guidelines and regulations. All studies were approved by the Ethics Committee for Animal Research at the University of Navarra and the Government of Navarra. *Collagen1α1-GFP* mice were previously described^17^.

### Induction of MI in mice

MI was induced in mice by ligation of the left anterior descending (LAD) coronary artery as previously described^18^. Briefly, 8 to 10 weeks old mice were anesthetized with vaporized isoflurane, intubated using a 20 G intravenous catheter, mechanically ventilated, and placed on a heating pad to maintain body temperature. A left thoracotomy was performed at the fourth-fifth intercostal space, where muscles were dissected. The LAD coronary artery was permanently ligated using a 7/0 non-absorbable ethylene suture. After visual verification of anemia and akinesis of the apex and anterior-lateral wall to ensure coronary occlusion, the thorax was closed in layers. After extubating, mice were kept warm until fully recovered. Healthy or infarcted mice at 1 to 7 dpi were sacrificed and processed for analyses.

### Single cell optimization and preparation

Mouse cardiac interstitial cells (CIC) from days 3 and 5 post-infarct were obtained from individual, 8–10-week-old mice as previously described^18^. Briefly, after euthanasia the thorax was opened and the heart was perfused with ice-cold phosphate buffered saline pH 7.6 (PBS) (Lonza), atria were excised and discarded, and both ventricles were digested completely. Excised tissues were placed in ice cold DMEM medium (Sigma) supplemented with 10% Fetal Bovine Serum (FBS) (Hyclone, GE). Ventricles were minced using a sterile scalpel. Pieces of tissue were incubated on an orbital shaker for 10 min at 37 °C in the presence of Liberase TH (125 µg/mL) (Roche) in HBSS +  + solution (Hanks balanced salt solution, Gibco). After enzymatic incubation, partially digested tissue was mechanically dissociated by slowly pipetting to generate a single cell suspension. The supernatant was filtered through a cell strainer to discard cardiomyocytes (CM) (40 μm, nylon; Falcon). The digestion was repeated with the sedimented pieces, and the supernatants were pooled together. Erythrocytes were removed using RBC lysis buffer (eBioscience). The total time for enzymatic digestion was 30 min.

In order to include all CFs in the following studies, an optimization step was performed as described previously^6^. Briefly, total CIC pellet was resuspended in 80 µL sorting buffer (2 mM EDTA, 0.5% BSA in PBS) and incubated with 20 µL of Feeder Removal MicroBeads (mEFSK4) (Miltenyi) for 15 min at 4 °C. A positive selection of CIC, enriched in CFs, was performed twice using LS columns (Miltenyi) according to the manufacturer’s instructions.

### Cell sorting

The positive fraction from the LS column enrich in CFs was centrifuged at 1,200 rpm and the pellet was resuspended in 100 µL of sorting buffer. Cells were incubated with the corresponding antibodies for 15 min at room temperature in the dark (Table 1). After incubation, the samples were washed twice with sorting buffer and spun at 1,200 rpm for 5 min, supernatant was discarded, and the final pellet was resuspended in 250 µL sorting buffer. Cell sorting was performed using FACSAria (BD Biosciences) and analyzed with FACSdiva software (BD Biosciences). Standard, strict forward scatter width *versus* area criteria were used to discriminate doublets and gate only singleton cells. Viable cells were identified by staining with 7-AAD (BD Bioscience). Viable cells gated on the FSC/SCC were sorted based on the expression of GFP (GFP^+^/CD31^−^/CD45^−^), and/or staining with anti-feeder cells antibody (mEFSK4 clone) (Miltenyi).

**Table 1:** List of antibodies used for cell sorting (FACS).

Name	Clone	Supplier	Dilution
CD31 (PECAM)-APC	MEC 13.3	BD Pharmingen, 551262	1:200
CD45-PE	30-F11	eBioscience 12-0451-81	1:200
Anti-feeder cells-APC	mEFSK4	Miltenyi, 130-102-302	1:50

### Bulk MARS-seq

Bulk RNA-seq was performed following a massively parallel RNA single-cell sequencing (MARS-seq) protocol adapted for bulk RNA-seq.^19,20^ with minor modifications. Briefly, 3,000 to 10,000 cells were sorted in 100 µL of Lysis/Binding Buffer (Ambion), vortexed and stored at −80 °C until further processing. Poly-A RNA was extracted with Dynabeads Oligo (dT) (Ambion) and reverse-transcribed with AffinityScript Multiple Temperature Reverse Transcriptase (RT) (Agilent) using poly-dT oligo primers (IDT) carrying a 7 bp index. Up to 8 samples with similar overall RNA content were pooled together and subjected to linear amplification via IVT using the HiScribe T7 High Yield RNA Synthesis Kit from New England Biolabs (NEB). The resulting antisense RNA was fragmented into 250–350 bps fragments with RNA Fragmentation Reagents (Ambion) and dephosphorylated with 1U FastAP (Thermo Scientific) for 15 min at 37 °C. Partial Illumina adaptor sequences^19^ were ligated with T4 RNA Ligase 1 (NEB) followed by a second reverse transcription. Full Illumina adaptor sequences were added during library amplification with KAPA HiFi DNA Polymerase (Kapa Biosystems). Libraries were quantified using a Qubit 3.0 Fluorometer and their size profiles examined in Agilent’s 4200 TapeStation System. Sequencing was carried out in an Illumina NextSeq500 using paired-end, dual-index sequencing (Read1: 68 cycles; Read2: 15 cycles; i7 index: 8 cycles) at a depth of 10 million reads per sample. Between 2 and 4 biological replicates per population were performed.

### Truseq RNA-sequencing

Total RNA from frozen biopsies of human hearts was isolated using TRIzol reagent (Ambion). Following mechanical homogenization with an Ultra-turrax (T10 basis Ultra-Turrax, IKA), RNA was either extracted according to the manufacturer’s instructions or stored at −80 °C until processed. RNA concentration was quantified using a Qubit 3.0 Fluorometer and its quality was examined in Agilent’s 4200 TapeStation System. RNA was subjected to rRNA depletion using Truseq Stranded Total RNA library prep Gold kit (Illumina), followed by RT, second strand synthesis, 3′ adenylation, Y shaped adaptor ligation and library enrichment as described above. Final libraries were quantified, and their profiles examined as described above. Sequencing was performed in an Illumina NextSeq500 using single-end, dual-index sequencing (Rd1: 75 cycles; i7: 8 cycles; i5: 8 cycles) at a minimum depth of 20 million reads per sample (n = 21).

### Single-cell RNA-sequencing (scRNA-seq)

The transcriptome of isolated GFP^+^-CFs from 8–12 weeks old, healthy or infarcted mice (3-, 5- or 7-dpi) were examined using Single Cell 3′ Reagent Kits v2 (10X Genomics) according to the manufacturer’s instructions. Two hearts were pooled in each scRNA-seq experiment. Briefly, 25,000 GFP^+^/CD31^−^/CD45^−^ or mEFSK4^+^/CD31^−^/CD45^−^ events were sorted in 1X PBS, 0.05% BSA and the number of cells was quantified in a Neubauer chamber. Approximately 16,000 cells were loaded at a concentration of 1,000 cells/µL on a Chromium Controller instrument (10X Genomics) to generate single-cell gel bead-in-emulsions (GEMs). In this step, each cell was encapsulated with primers containing a fixed Illumina Read 1 sequence, followed by a cell-identifying 16 nt 10X barcode, a 10 nt Unique Molecular Identifier (UMI) and a poly-dT sequence. Upon cell lysis, reverse transcription yielded full-length, barcoded cDNA. This cDNA was then released from the GEMs, PCR-amplified and purified with magnetic beads (SPRIselect, Beckman Coulter). Enzymatic Fragmentation and Size Selection was used to optimize cDNA size prior to library construction. Fragmented cDNA was then end-repaired, A-tailed and ligated to Illumina adaptors. A final PCR-amplification with barcoded primers allowed sample indexing. Library quality control and quantification was performed using Qubit 3.0 Fluorometer (Life Technologies) and Agilent’s 4200 TapeStation System (Agilent), respectively. Sequencing was performed in a NextSeq500 (Illumina) (Read1: 26 cycles; Read2: 57 cycles; i7 index: 8 cycles) at an average depth of 50,000 reads/cell.

### Tissue processing and Visium data generation

The hearts from *Collagen1α1-GFP* 8–12 weeks old, healthy or infarcted mice (3 and 5 dpi) were excised and washed in PBS, embedded in OCT compound, frozen in dry ice and sectioned in a cryostat at a thickness of 10 μm at −20 °C following the manufacturer’s recommendations.

In order to check the RNA quality of the tissues, ten tissue sections of each sample were collected in an RNase-free Eppendorf tube, and RNA was extracted with the Qiagen RNeasy extraction kit (Qiagen) according to the manufacturer’s instructions. RNA quality was assessed with a 4200 TapeStation (Agilent Technologies), and all the samples had a RIN between 7.2 and 7.7.

For optimization of the tissue permeabilization, seven sections from the 3-dpi sample were collected onto a −20 °C pre-equilibrated Visium tissue optimization slide (10X Genomics) as per the manufacturer’s recommendations. Briefly, tissue sections were fixed in chilled methanol, then stained with Mayer’s Hematoxylin solution (Millipore Sigma), followed by Bluing Buffer (Dako), and Eosin Y solution (Millipore Sigma). Hematoxylins & Eosin (H&E) stained tissues were imaged on an Aperio CS2 (Leica Biosystems) digital slide scanner at 40X magnification. Tissues on optimization slides were permeabilized in a time course experiment from 3 to 30 minutes, and reverse transcription was performed using fluorescently labeled nucleotides, resulting in fluorescent cDNA bound to the capture areas within the slide. Tissues were enzymatically removed, and fluorescence imaging was performed using a Zeiss LSM800 (Carl Zeiss Microscopy) digital slide scanner with a red filter set. The whole slide scan was carried out using a 20X magnification objective with 150 milliseconds exposure per image frame. Sequentially imaged frames were automatically stitched. A permeabilization time of 12 minutes resulted in the maximum fluorescence signal with the lowest signal diffusion and was chosen as the optimal time to perform the subsequent Visium expression experiments.

In the final experiment, the mRNA capture was performed following instructions of the Visium Spatial Gene Expression Reagent Kits User Guide (10x Genomics). Briefly, one section of each sample was collected onto a pre-equilibrated Visium expression slide. After H&E staining, stained tissues were imaged on the Aperio CS2 (Leica Biosystems) digital slide scanner at 40X magnification. The sections were permeabilized for 12 minutes to release poly-adenylated mRNA from overlying cells onto the capture areas of the slide. The bound mRNAs were then reverse transcribed, resulting in spatially barcoded, full-length cDNA. Second strand synthesis was performed, followed by denaturation and transfer of the cDNA from the slide to a PCR tube. qPCR (KAPA SYBR FAST qPCR Master Mix, KAPA Biosystems) was used to determine the number of cDNA amplification cycles required based on the 25% RFU peak. After 16 cDNA amplification cycles, fragment analysis of the cDNA was performed on a 4200 TapeStation (Agilent Technologies). Library construction, performed on 25% of the cDNA, consisted of enzymatic fragmentation of the cDNA, end-repair, A-tailing and adaptor ligation, followed by a sample index PCR. Final libraries containing P5 and P7 primers were quantified with Qubit 3.0 Fluorometer (Life Technologies). Libraries were loaded at 650 pM and sequenced on a Nextseq. 2000 System (Illumina) using a Nextseq. 2000 P3 Reagent Kit (100 cycles, Illumina), at an average sequencing depth of approximately 100,000 reads per spot (25–35% of the 5,000 total spots on the slide were used). The protocol was performed using the following read protocol: Read1, 28 cycles; i7 index, 10 cycles; i5 index, 10 cycles; Read2, 91 cycles.

### Immunofluorescence

Adult mouse hearts were excised and washed in PBS, fixed in 4% fresh paraformaldehyde (Sigma). Some specimens were then cryoprotected in sucrose (Sigma), embedded in OCT compound (VWR), frozen in dry ice and stored until sectioning. For Reparative Cardiac Fibroblast (RCF) specific marker staining additional specimens were dehydrated, paraffin embedded, sectioned and rehydrated before the staining. Five-to-ten µm thick sections were washed in Tris-PBS (TPBS), non-specific IgG binding sites were blocked with 8% goat serum (Dako), 1% BSA, and 0.1% Triton X-100 (Sigma) followed by incubation overnight with the corresponding primary antibodies at 4 °C (Table 2). Anti-GFP staining required an antigen retrieval step using 100 mM citrate buffer pH6.3 (Sigma) before blocking. The sections were then washed and incubated with the appropriate fluorescence-conjugated secondary antibodies for 1 hour before mounting (Table 2). Negative controls were included by omitting the primary antibody. Cell nuclei were counterstained with 4′,6-diamidino-2-phenylindole (DAPI, Sigma). All images were captured in a Zeiss LSM 510 800 (Zeiss) or Leica SP5 confocal laser microcopy (Leica).

**Table 2:** List of antibodies used for immunofluorescence analyses.

Name	Clone	Supplier	Dilution
CTHRC1	Vli55	Maine Medical Ctr. Res. Institute	100 ng/ml
GFP	pAb	Abcam, ab13970	1:100
Asporin	pAb	Thermo Scientific, PA5112364	1:100
Anti-Chicken 488	2ry Ab	Jackson Inmunoresearch, 703-545-155	1:200
Anti-Rabbit 647	2ry Ab	Invitrogen, A31573	1:200

### RNA in situ hybridization (ISH) assay by RNAscope®

Mice adult hearts were fixed in formalin overnight at 4 °C and washed three times with PBS for 5 min. Then, samples were dehydrated, paraffin embedded and sectioned into 10 µm slides. Sections were analyzed with an RNAscope® assay, using the RNAscope® Multiplex Fluorescent Reagent Kit v2 (Advance Cell Diagnostics) following manufacturer’s instructions. Briefly, sections were dehydrated, deparaffinized, and incubated with hydrogen peroxide solution. Subsequently, sections were treated with RNAscope® Target Retrieval reagent solution, and then protease plus was applied in a humid chamber. Then, sections were incubated with the following targeted probes: *Postn* (ACD^TM^ Bio, 418581-C1), *Aspn* (ACD^TM^ Bio, 502051-C2), *Cthrc1* (ACD^TM^ Bio, 416641-C4), and RNAscope^TM^ 3-plex Negative Control probe (all from Advance Cell Diagnostics). The hybridization procedure was performed for 2 h at 40 °C, followed by an overnight incubation with 5X SSC buffer (20X SSC Buffer, Invitrogen) at room temperature. The next day, sections were incubated with the detection reagents for manual amplification and washed twice with the RNAscope® Wash Buffer. After amplification, sections were incubated subsequently with the appropriate HRP channel, the corresponding Opal (Akoya Bioscience) and the HRP blocker, with RNAscope® Wash Buffer between incubations. Finally, the sections were incubated with DAPI and mounted with ProLong^TM^ Gold antifade reagent (Invitrogen).

### Bulk RNA-seq analysis

Samples were demultiplexed using Illumina *bcl2fastq* software (1.2.4) and aligned with the mouse (*mm10*) or human (*GRCh38*) genome with STAR (2.6.1)^21^ setting the parameters to default values. Quantification and generation of gene expression matrices were performed with the function *featureCounts*, implemented in the R^22^ package *Rsubread*^23^. The *ensembl* transcriptomes (*GRCm38.91* and *GRCh38.92*) were used as reference for gene annotation. An initial filtering of not expressed genes was performed. Additional filtering, data transformation, normalization, and testing for differential expression was performed with DESeq2^24^.

For human samples, GSVA^25^ was used to compute scores for the expression of different gene signatures. We applied GSVA to the DESeq2 normalized expression matrix. We calculated scores for the following signatures: 1) Velocity signature; 2) gene cluster 4; 3) gene cluster 9; 4) the ratio between gene cluster 4 and 9.

### Single cell RNA-seq analysis

Sequenced libraries were demultiplexed, aligned to the mouse transcriptome (*mm10*) and quantified using *Cell Ranger* (3.0.1) from 10X Genomics. The output of the pre-processing pipeline consisted of gene expression matrices per cell. Further computational analysis was performed using *Seurat* (3.1.0). Cells were subjected to quality control filters based on the number of detected genes, number of UMIs and proportion of UMIs mapped to mitochondrial genes per cell. Regarding the number of genes and UMI, we established the first and fourth quartile as lower and upper threshold, respectively. Cells with >5% mitochondrial genes were also filtered out. Using these parameters, 6,277 (healthy myocardium), 7,888 (3 dpi), 4,839 (5 dpi), 8,991 (7 dpi) cells were retained.

Each single cell dataset was subjected to log normalization and scaling. Additional normalization and variance stabilization, together with removal of unwanted source of variation (% of mitochondrial genes and UMI numbers), was performed using the SCTransform method^26^.

Next, we integrated the 4 time points using a set of anchors that was found based on canonical correlation analysis (CCA)^27^. A total of 2,000 variable features were used as input for the analysis, and 30 canonical vectors were calculated.

The integrated single cell dataset was rescaled and subjected to principal component analysis (PCA). Unsupervised clustering was performed, using the 20 first PCs and a resolution parameter of 0.8. The optimal clustering resolution was determined based on Clustree visualization and Adjusted Rand Index (ARI) analysis. Non-linear dimensional reduction was computed using uniform manifold approximation and projection (UMAP)^28^.

Gene signature characterizing RCFs was obtained from Ruiz-Villalba *et al*.^6^. We selected the set of markers from cluster B with logFC > 0.1 and adjusted p value < 0.01. This signature was used as input for AUCell^29^, in order to explore its activity in the individual cells. Expression ranking was created with the normalized counts (after SCTransform). All differential expression tests were done using the function *FindAllMarkers* with the MAST test^30^.

### RNA Velocity

For the velocity analysis, we created a subset of the Seurat dataset selecting exclusively the cells expressing *Postn*. Every cell displaying *Postn* raw counts > 0 was considered *Postn*^+^ and included in the subset. We reprocessed this subset following the steps described above (log normalization, scaling, SCTransform and integration) and computed a new UMAP. After reprocessing, we used the new SCT corrected matrix as input for the velocity.

The spliced/unspliced matrices were generated with *velocyto*^31^ (0.17.17) for each condition (healthy, 3, 5 and 7 dpi) using the BAM files from *CellRanger* (3.0.1) as input. Further processing was performed using *scVelo*^32^ (version 0.2.3) implemented in *Python* (3.8.8). Data was preprocessed by filtering the top 2000 highly variable genes, PCA and neighborhood graph construction, using default options. The dynamical model was used to estimate RNA velocities. The stream plots were projected on the UMAP reductions retrieved with *Seurat*. Differential expression analysis was applied to the gene velocities, using the function *rank_velocity_genes*, to rank genes based on differential dynamics across cell clusters. Genes resulting from this analysis were finally subjected to hierarchical clustering using their ranking positions as input values.

### Spatial gene expression analysis

Visium libraries were demultiplexed and mapped with *Space Ranger* (1.3.1) from 10X Genomics. Libraries were mapped to an Ensembl 105 based reference (GRCh39 reference, v3.0.0). The reference was modified to include the GFP gene, following 10X instructions. Manual alignment was performed with *Loupe Browser* (6.1.0) to select spots under the tissue. Spatial transcriptomic data was processed using *Seurat*^27^ (4.1.1). Quality control was performed in order to remove spots with low quality defined as UMI < 500, gene < 250 and mitochondrial (%) > 50. After, we normalized data using the regularized negative binomial regression implemented in the *sctranform*^26^ R-package and a visual exploration was performed through PCA, unsupervised shared-nearest-neighbor-based clustering and UMAP. Enrichment analysis for predefined signatures (cluster B/RCFs, gene cluster 4 and 9 signatures) was performed using *AUCell*^29^ (1.16.0) for each spot in the samples. After this step, we adapted a published criterion^33^ to identify anatomical domains based on cardiomyocyte transcriptomic profiles, dividing the heart tissue samples spots on remote zone (RZ), border zones 1 (BZ1, closer to RZ), border zone 2 (BZ2, closer to the infarct zone/IZ), and infarct zone (IZ). Enrichment score was then plotted by segment to understand the score gradient in the RCFs, which included cluster 5, 8, 9 and 11, and the score in dynamics 1 and 2 and the ratio between them. Linear regression was performed per spot with *stats*^22^ (3.6.2) to study the dependence of the signature’s gradient with the fibroblast population in the different areas of the tissue.

## Data Records

Single-cell RNA-seq data from experiments performed for this study, 3 and 5 dpi, are available at NBCI’s Gene Expression Omnibus database under accession number GSE261428^34^. Bulk RNA-seq generated to define the optimal time points, is available at NBCI’s Gene Expression Omnibus database under accession number GSE267256^35^. Spatial transcriptomics are available at NBCI’s Gene Expression Omnibus database under accession number GSE265828^36^.

Single-cell RNA-seq on healthy, and 7 dpi are available at NBCI’s Gene Expression Omnibus database under accession number GSE132146^16^, samples GSM3847600 and GSM3847601. Bulk RNA-seq samples on control, 5, 7, 14 and 30 dpi used, are available at NBCI’s Gene Expression Omnibus database under the accession number GSE132146 SuperSeries^16^, samples from GSM3847463 to GSM3847476, and GSM3847563, and GSM3847564.

RNA-seq from infarcted pig hearts is available at NBCI’s Gene Expression Omnibus database under the accession number GSE132146 SuperSeries^16^, samples labelled “Swine” (from GSM4760658 to GSM4760669, and from GSM3847571 to GSM3847596). RNA-seq from human biopsies samples collected from the RZ and IZ of patients with ischemic cardiomyopathy is available at NBCI’s Gene Expression Omnibus database under the accession number GSE132146 SuperSeries^16^, samples labelled “MI Patient” (from GSM3847567 to GSM3847570, and from GSM4141391 to GSM4141406) and “Healthy” (GSM3847565, GSM3847566, and from GSM4760638 to GSM4760647).

## Technical Validation

### *Cthrc1*^+^ Reparative Cardiac Fibroblasts arise from *Postn*^+^ Activated Cardiac Fibroblasts

We addressed the biological value of the single-cell dataset to elucidate the potential origin of *Cthrc1*^+^ RCFs (Fig. 2a). A total of 27,995 CFs were grouped in 17 different cell clusters (Fig. 2b). Second, we identified the clusters that contain activated CFs based on the expression of Periostin (*Postn*). In healthy cardiac tissue, *Postn*^+^ CFs were mostly found in cluster 9. After MI, the number of these cells increased in almost every cluster (Fig. 2c). In contrast, the first RCFs were observed after infarct in clusters 5, 8, and 11, and increased their number onwards (Fig. 2d). These three cell clusters showed the highest number of *Cthrc1*^+^ cells (Fig. 2e), together with the major similarities with the transcriptomic profile (Fig. 2f), and number of RCF genes (Fig. 2g). The concordant enrichment of *Postn* and *Cthrc1* expression across defined clusters and time points supports the robustness of the clustering strategy and the reliability of cell-state annotation within the dataset.

### Spatial dynamics of the transition into *Cthrc1*^+^ Reparative Cardiac Fibroblasts

**Fig. 3:**
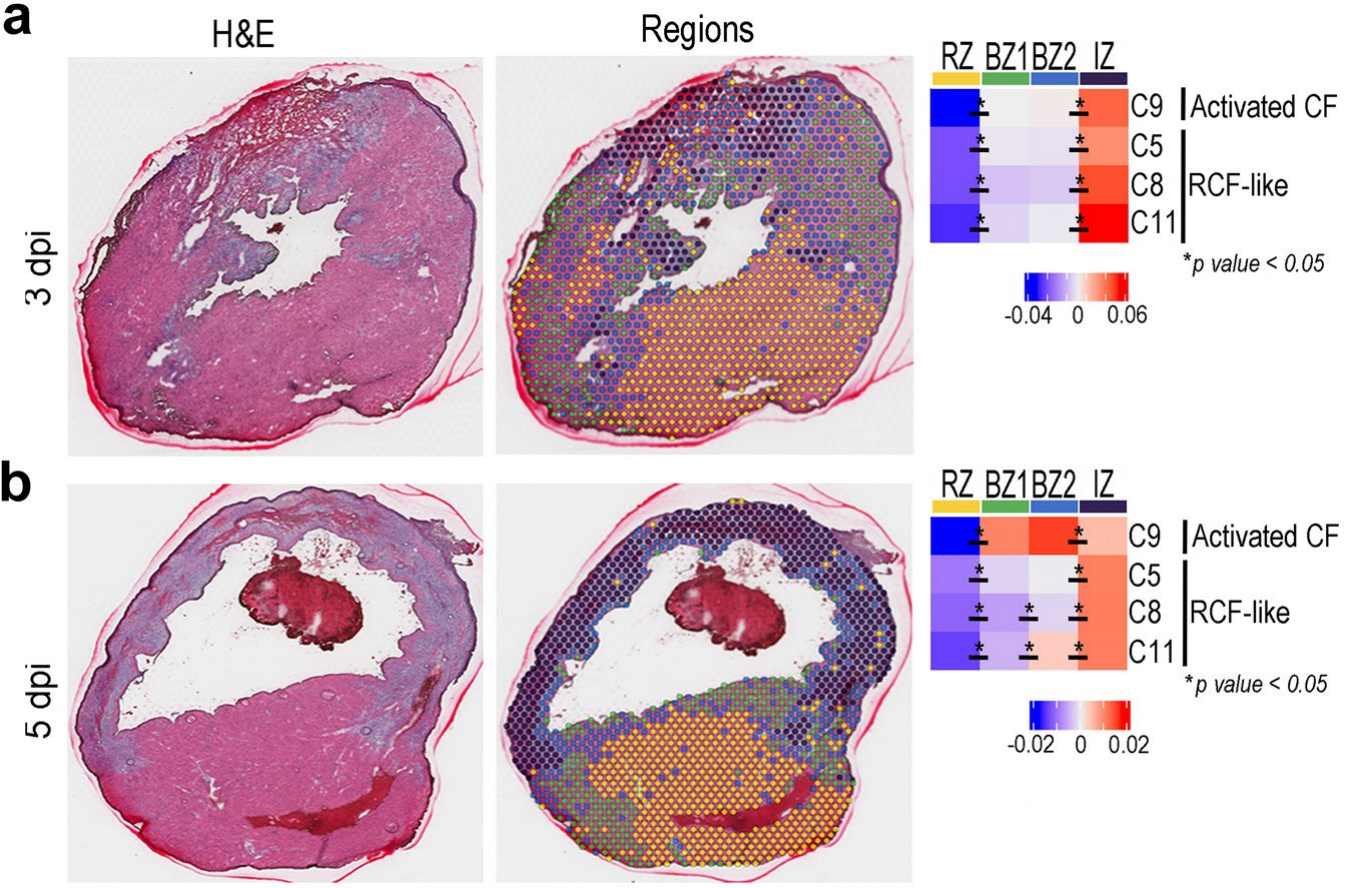
Spatial transcriptomics location of different CF clusters. Spatial transcriptomics (10x Genomics Visium) of transverse heart sections at 3 (**a**) and 5 dpi (**b**). H&E-stained sections were segmented into infarct zone (IZ) (purple), remote zone (RZ) (yellow), and two border zones [BZ1 adjacent to RZ (blue), and BZ2 adjacent to IZ (green)], based on cardiomyocyte transcriptional profiles^33^. Heatmap shows average gene set scores per spot for clusters 5, 8, 9, and 11. *P value* < 0.05 for all the comparisons indicated with *.

We investigated the value of the spatial profiling to disentangle the ventricular distribution of *Postn*^+^*/Cthrc1*^*−*^ versus *Postn*^+^*/Cthrc1*^+^ CFs using spatial transcriptomics. To achieve this goal in an unbiased manner, we first adapted a previously published criterion to identify anatomical domains based on cardiomyocyte transcriptomic profiles^33^. Our approach identified four different domains on infarcted heart tissue samples: remote zone (RZ), border zone 1 (BZ1, closer to RZ), border zone 2 (BZ2, closer to the infarct zone/IZ), and IZ (Fig. 3a). Such domains tightly correlate with the histomorphology traits observed after hematoxylin-eosin staining on the same tissue sections. Spatial enrichment analysis demonstrated that signatures corresponding to both *Postn*^+^ */Cthrc1*^−^ and *Postn*^+^ */Cthrc1*^+^ CFs are enriched in the IZ at 3 dpi. At 5 dpi, however, this up-regulation in IZ is more specific for RCFs. Moreover, the RCF transcriptional signature is gradually enriched, following a characteristic gradient from the RZ to the IZ (Fig. 3b). The reproducible alignment of fibroblast-associated gene signatures with transcriptomically and histologically defined anatomical zones supports the spatial resolution, annotation strategy, and biological coherence of the dataset.

### Characterizing the transition from activated to reparative cardiac fibroblast

**Fig. 4:**
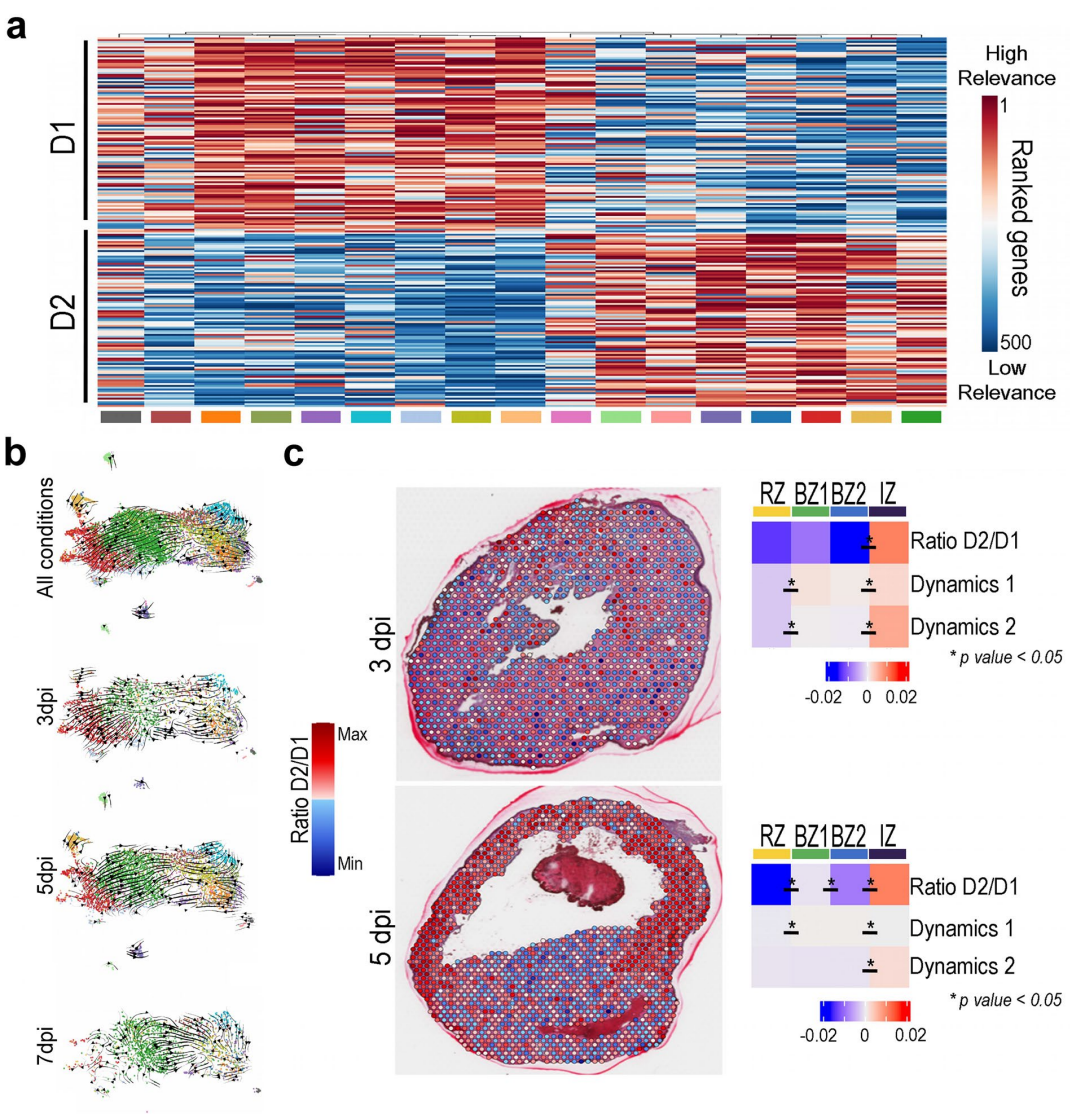
Temporal and anatomical gene expression dynamics of CF transitional states during myocardial repair. (**a**) Heatmap showing the top ranked genes obtained in the RNA velocity analysis with significant contributions to cellular trajectories in *Postn*^+^ CFs within the first week after MI. A total of 600 genes were identified and grouped into two transcriptional programs: dynamics 1 (D1) and dynamics 2 (D2) (Supplementary Excel file). (**b**) UMAP plots illustrate cellular trajectories of *Postn*^+^ CFs within all the CF clusters at the individual time points studied, based on splicing kinetics of RCF signature genes. (**c**) Spatial transcriptomics representations showing the D1 score, D2 score, and D2/D1 ratio across spatial regions at 3 and 5 dpi. *p* < 0.05.

Next, we investigated whether the dataset enabled the identification of potential global transcriptional dynamics underlying the transition from activated CFs (*Postn*^+^*/Cthrc1*^*−*^) to RCFs (*Postn*^+^*/Cthrc1*^+^). To that end, we first selected activated CFs and RCFs cells for each cluster, and we performed RNA velocity analysis using *velocito*^32^. As a result, we obtained a ranked list of genes for each cluster, reflecting their contributions to the observed differentiation trajectories. We then compared these rankings across all clusters, which revealed two distinct gene groups and their associated cell clusters. These were defined as two putative transcriptional dynamics, termed D1 and D2 (Fig. 4a and Supplementary Excel file). Notably, only D2 displayed a branching structure that culminated in a terminal trajectory toward RCFs. Consistent with this, the D2-associated trajectory was no longer detectable by 7 dpi, at which point a single cell cluster remained that retained the transcriptomic signature of RCFs (Fig. 4b). Spatial transcriptomic analysis revealed a statistically significant enrichment of D2-associated gene expression in the infarct zone (IZ) at both 3 and 5 dpi (Fig. 4c), highlighting the spatial specificity of this transcriptional program. The concordance between velocity-derived gene dynamics, temporal resolution, and spatial localization supports the robustness of trajectory inference and the internal consistency of the dataset across analytical modalities.

### Validating a potential master gene regulator in the switch into RCFs

**Fig. 5:**
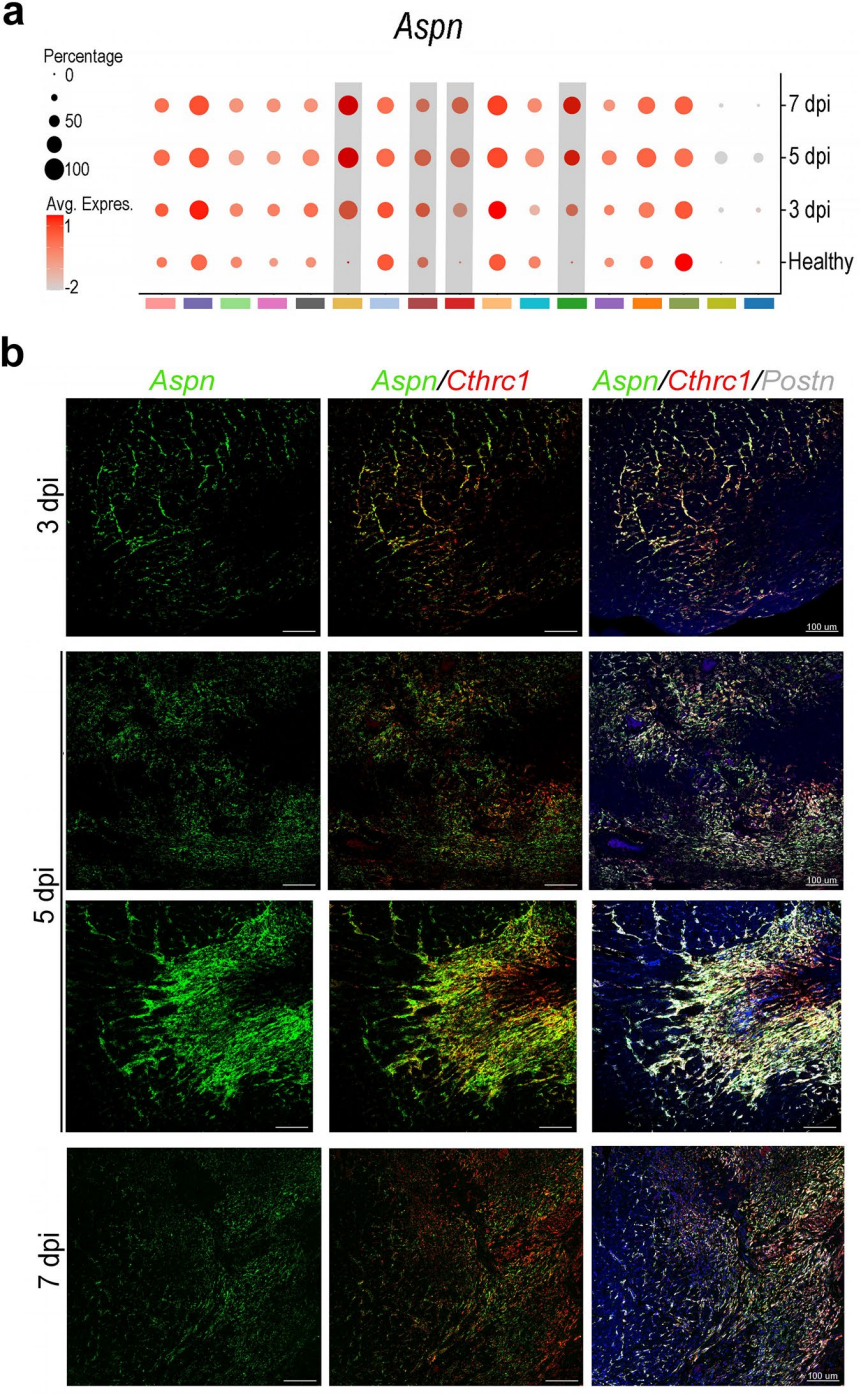
Temporal and anatomical gene expression dynamics of Asporin during early MI. (**a**) Dot plot showing *Aspn* expression enrichment across all CF clusters and time points. Dot size indicates the percentage of expressing cells; color reflects normalized expression level. Grey bars highlight RCF-like clusters. (**b**) Representative RNAscope images of Asporin (*Aspn*, green), Collagen triple helix repeat containing 1 (*Cthrc1*, red), and Periostin (*Postn*, white) mRNA in transverse sections of murine hearts at 3, 5, and 7 dpi.

**Fig. 6:**
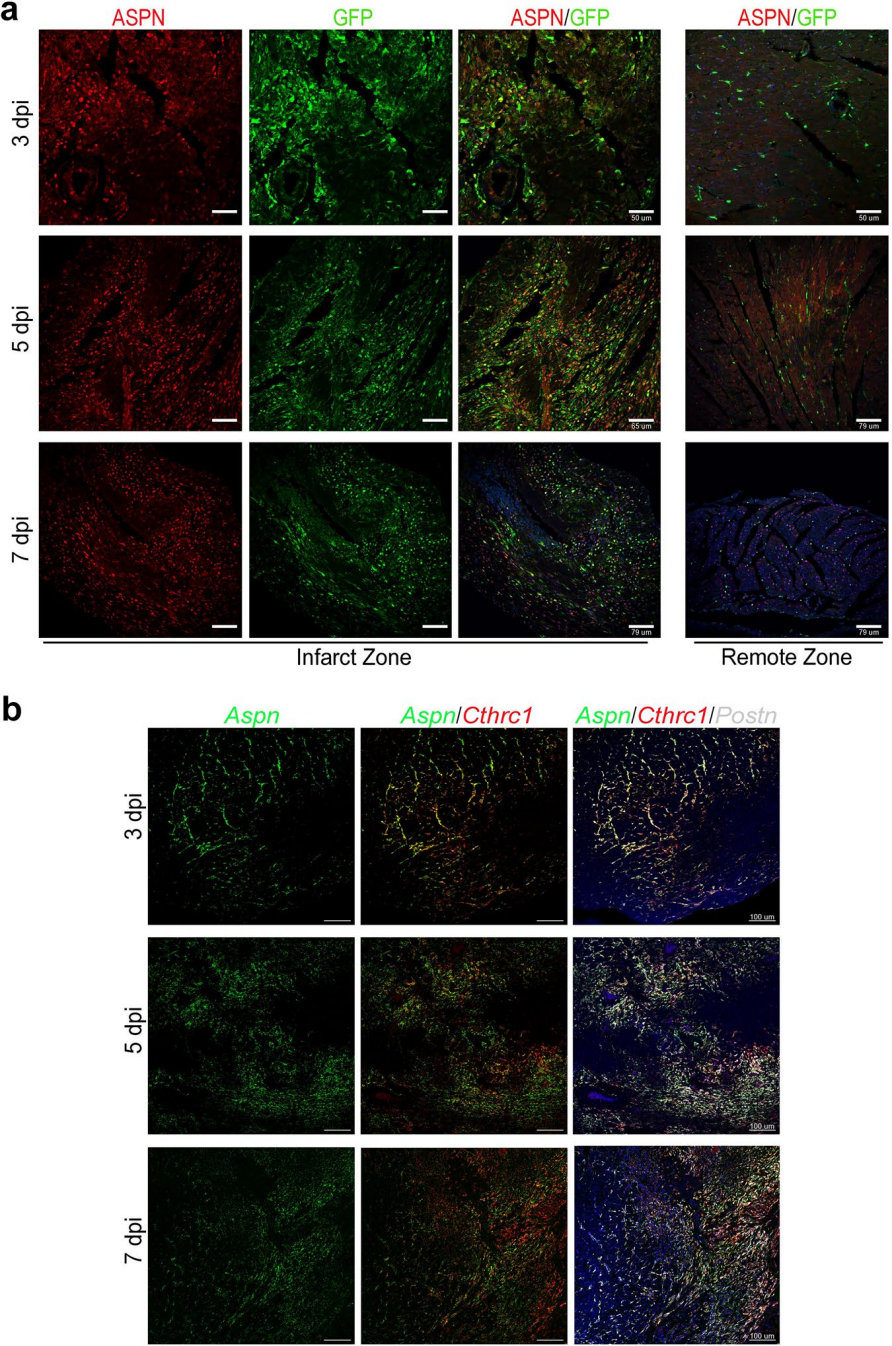
Immunohistochemistry analysis of RCF marker genes in infarcted mouse hearts. Representative immunohistochemistry of ASPN (red) in the infarct and remote zones of *Col1α1*-*GFP* reporter mouse hearts at 3, 5, and 7 dpi. GFP-positive CFs appear green, nuclei are stained with DAPI (blue), and signal colocalization appears yellow.

Next, to confirm the value of the dynamics uncovered in the single-cell dataset we investigated the D2 dynamics. We observed that D2 genes were associated with the “TGF-β signaling pathway”, and extracellular matrix molecules, such as *Fndc1, Ltbp3*, or *Aspn*. This annotation supports the coherence of the D2 gene set with established ECM–associated transcriptional programs observed in post-infarction CFs. Among D2 genes, we identified Asporin (*Aspn*) as a suitable requisite for RCF appearance based on different criteria. First, ASPN is an ECM protein that acts as a natural inhibitor of the canonical “TGF-β signaling pathway”. Second, *Aspn* loss-of-function yields a *Cthrc1*-KO-like phenotype of ventricular rupture phenotype at 5 dpi in mice^37^. Third, *Aspn* expression is upregulated in post-MI CFs when compared with healthy ones in our analysis (Fig. 5a). When *Aspn* expression was evaluated on infarcted heart tissues using RNAscope, we observed a gradual transcriptional shift from *Postn*^*+*^*/Aspn*^*+*^*/Cthrc1*^*−*^ activated CFs, at the most external region of the BZ, to *Postn*^*+*^*/Aspn*^*−*^*/Cthrc1*^*+*^ RCFs, in the most internal area of the fibrosis along the different analyzed time points (Fig. 5b). Accordingly, an increase of ASPN^+^ CFs was detected from 3 to 7 dpi in the border and infarcted zones of the infarcted hearts (Fig. 6). The agreement between single-cell transcriptomic signatures, spatial transcriptomics, and independent *in situ* hybridization supports the robustness of the inferred D2-associated gene dynamics and the reliability of marker-based validation across experimental modalities.

### Translational validation

**Fig. 7:**
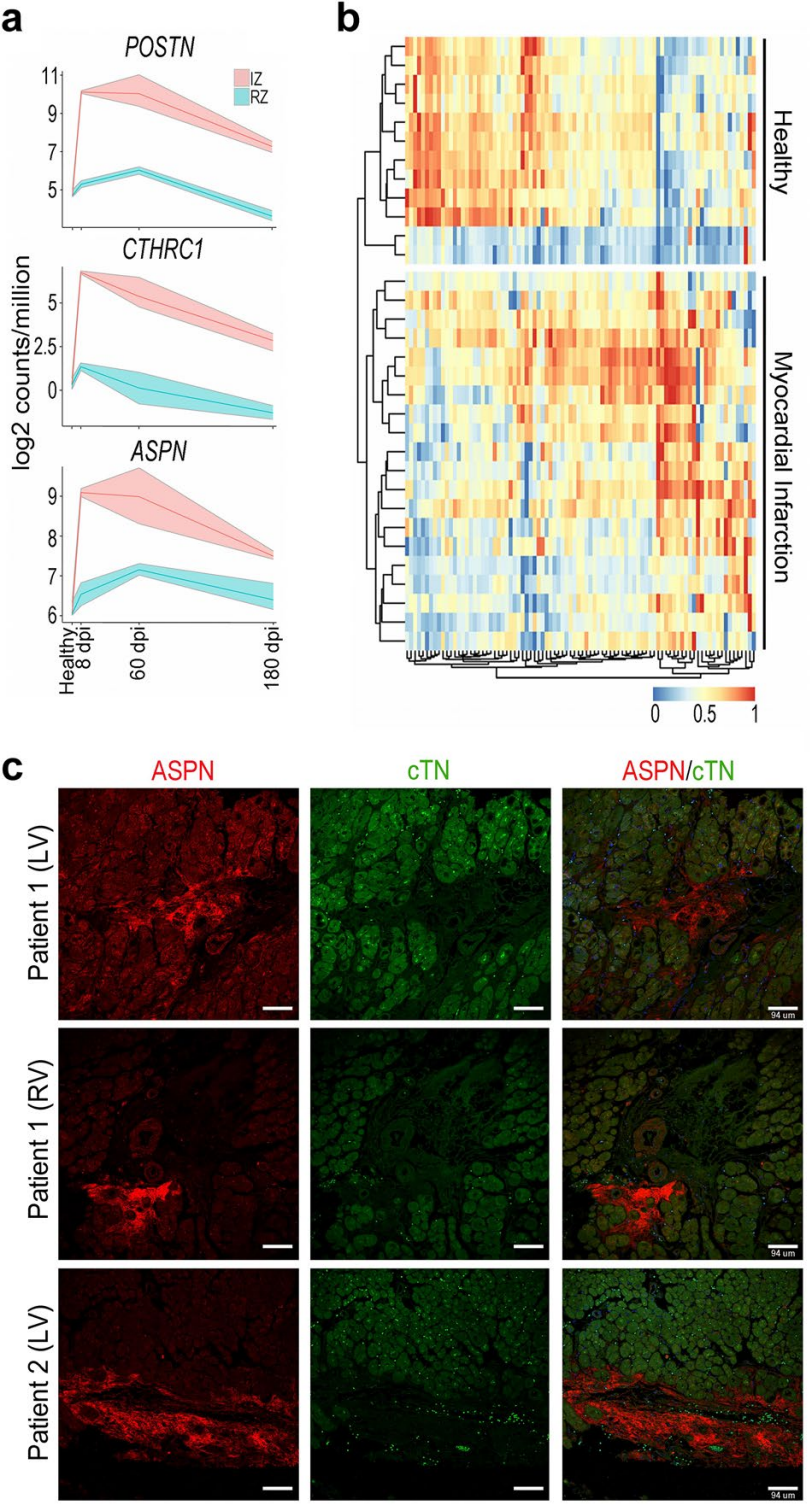
Conservation of the dynamics of Asporin expression in large-animal models of MI and human infarcted hearts. (**a**) Expression of *ASPN*, *CTHRC1*, and *POSTN*, analyzed in a previously published dataset, in infarct zone (IZ) and remote zone (RZ) of porcine (*Sus scrofa*) hearts at 0, 8, 60, and 180 dpi^6^. Data are log^2^-normalized counts per million. Lines represent means; shaded ribbons indicate interquartile ranges. (**b**) Heatmaps showing D2 gene set expression in bulk RNA-seq from healthy donor hearts and infarcted patient hearts^6^. (**c**) Representative immunohistochemistry showing *ASPN* (red) and *cTN* (green, cardiomyocytes) expression in heart biopsies from infarcted patients.

To evaluate cross-species consistency and external reproducibility of the transcriptional patterns identified in the dataset, publicly available large-animal and human cardiac transcriptomic data were analyzed. Expression of *ASPN* was first examined in myocardial biopsies from infarcted pig hearts using the GSE132146 SuperSeries dataset (GSE132146 SuperSeries)^16^ (Fig. 7a). We observed a temporal window of *ASPN* up-regulation in the infarcted ventricle (IZ), which correlates with the expression of both *POSTN* and *CTHRC1*, supporting concordant regulation of these markers in a large-animal model. In parallel, we studied the transcriptional profile of human biopsy samples collected from the heart of healthy patients and from IZ of patients with ischemic cardiomyopathy (GSE132146 SuperSeries)^16^. This approach identified a subset of D2 genes that are significantly upregulated within MI samples (Fig. 7b), results that were validated at proteomic level in two different patients (Fig. 7c). The reproducible detection of D2-associated gene expression patterns across mouse, pig, and human datasets, as well as across bulk transcriptomic, single-cell, spatial, and proteomic platforms, supports the robustness, interoperability, and reuse potential of the dataset for comparative and translational cardiac research.

In summary, this study provides a comprehensive single-cell and spatial transcriptomic dataset capturing CF heterogeneity during early post–MI remodeling. The dataset integrates single-cell, spatial, *in situ*, and cross-species transcriptomic analyses, enabling the systematic characterization of CF subpopulations and their temporal and spatial distributions between 3 and 5 dpi. Cross-modal and cross-species concordance, including consistent detection of *ASPN*-associated transcriptional signatures, supports the robustness and reproducibility of the data.

By combining orthogonal validation strategies and publicly available large-animal and human datasets, this resource offers a well-annotated reference framework for studying fibroblast-associated transcriptional programs during cardiac injury. This dataset is expected to facilitate comparative analyses, methodological benchmarking, and hypothesis generation in the cardiac fibrosis research field, and may be broadly applicable to studies of fibroblast biology and tissue remodeling across organs.

## Supplementary information

Supplementary Excel file containing the lists of genes within Dynamic1 and Dynamic 2 determined by their roles in dynamic transcriptional shifts revealed by RNA velocity analysis and a ranking strategy.

## Data Availability

We confirm that the data is publicly available in the NBCI’s Gene Expression Omnibus database under accession numbers: GSE261428^34^, GSE267256^35^, GSE265828^36^, and GSE132146^16^.

## Code availability

All script for the analysis used to generate the processed data and the visualizations/figures in the present manuscript are available on https://github.com/lsudupe/Cardio_paper/.

## References

[1] Thannickal, V. J., Zhou, Y., Gaggar, A. & Duncan, S. R. Fibrosis: ultimate and proximate causes. *J Clin Invest* **124**, 4673–4677, 10.1172/JCI74368 (2014). 25365073 10.1172/JCI74368 PMC4347226

[2] Janbandhu, V. *et al*. Novel Mouse Model for Selective Tagging, Purification, and Manipulation of Cardiac Myofibroblasts. *Circulation* **149**, 1931–1934, 10.1161/CIRCULATIONAHA.123.067754 (2024). 38857329 10.1161/CIRCULATIONAHA.123.067754

[3] Tsukui, T., Wolters, P. J. & Sheppard, D. Alveolar fibroblast lineage orchestrates lung inflammation and fibrosis. *Nature*, 10.1038/s41586-024-07660-1 (2024). 10.1038/s41586-024-07660-1 PMC12088911 38987592

[4] Kuppe, C. *et al*. Decoding myofibroblast origins in human kidney fibrosis. *Nature* **589**, 281–286, 10.1038/s41586-020-2941-1 (2021). 33176333 10.1038/s41586-020-2941-1 PMC7611626

[5] Kuppe, C. *et al*. Spatial multi-omic map of human myocardial infarction. *Nature* **608**, 766–777, 10.1038/s41586-022-05060-x (2022). 35948637 10.1038/s41586-022-05060-x PMC9364862

[6] Ruiz-Villalba, A. *et al*. Single-Cell RNA Sequencing Analysis Reveals a Crucial Role for CTHRC1 (Collagen Triple Helix Repeat Containing 1) Cardiac Fibroblasts After Myocardial Infarction. *Circulation* **142**, 1831–1847, 10.1161/CIRCULATIONAHA.119.044557 (2020). 32972203 10.1161/CIRCULATIONAHA.119.044557 PMC7730974

[7] Driskell, R. R. *et al*. Distinct fibroblast lineages determine dermal architecture in skin development and repair. *Nature* **504**, 277–281, 10.1038/nature12783 (2013). 24336287 10.1038/nature12783 PMC3868929

[8] Yang, W. *et al*. Single-Cell Transcriptomic Analysis Reveals a Hepatic Stellate Cell-Activation Roadmap and Myofibroblast Origin During Liver Fibrosis in Mice. *Hepatology* **74**, 2774–2790, 10.1002/hep.31987 (2021). 34089528 10.1002/hep.31987 PMC8597108

[9] Amrute, J. M. *et al*. Targeting immune-fibroblast cell communication in heart failure. *Nature* **635**, 423–433, 10.1038/s41586-024-08008-5 (2024). 39443792 10.1038/s41586-024-08008-5 PMC12334188

[10] Kleinbongard, P. *et al*. Cardiac fibroblasts: answering the call. *Am J Physiol Heart Circ Physiol* **327**, H681–H686, 10.1152/ajpheart.00478.2024 (2024). 39093000 10.1152/ajpheart.00478.2024 PMC11442096

[11] Hilgendorf, I., Frantz, S. & Frangogiannis, N. G. Repair of the Infarcted Heart: Cellular Effectors, Molecular Mechanisms and Therapeutic Opportunities. *Circ Res* **134**, 1718–1751, 10.1161/CIRCRESAHA.124.323658 (2024). 38843294 10.1161/CIRCRESAHA.124.323658 PMC11164543

[12] Rieder, F. *et al*. Fibrosis: cross-organ biology and pathways to development of innovative drugs. *Nat Rev Drug Discov*10.1038/s41573-025-01158-9 (2025). 40102636 10.1038/s41573-025-01158-9 PMC13264708

[13] Konkimalla, A. *et al*. Transitional cell states sculpt tissue topology during lung regeneration. *Cell Stem Cell* **30**, 1486–1502 e1489, 10.1016/j.stem.2023.10.001 (2023). 37922879 10.1016/j.stem.2023.10.001 PMC10762634

[14] Li, J. *et al*. Autocrine CTHRC1 activates hepatic stellate cells and promotes liver fibrosis by activating TGF-beta signaling. *EBioMedicine* **40**, 43–55, 10.1016/j.ebiom.2019.01.009 (2019). 30639416 10.1016/j.ebiom.2019.01.009 PMC6412555

[15] Buechler, M. B. *et al*. Cross-tissue organization of the fibroblast lineage. *Nature* **593**, 575–579, 10.1038/s41586-021-03549-5 (2021). 33981032 10.1038/s41586-021-03549-5

[16] *NCBI Gene Expression Omnibus* https://identifiers.org/geo/GSE132146 (2020).

[17] Yata, Y. *et al*. DNase I-hypersensitive sites enhance alpha1(I) collagen gene expression in hepatic stellate cells. *Hepatology* **37**, 267–276, 10.1053/jhep.2003.50067 (2003). 12540776 10.1053/jhep.2003.50067

[18] Ruiz-Villalba, A. *et al*. Interacting resident epicardium-derived fibroblasts and recruited bone marrow cells form myocardial infarction scar. *J Am Coll Cardiol* **65**, 2057–2066, 10.1016/j.jacc.2015.03.520 (2015). 25975467 10.1016/j.jacc.2015.03.520

[19] Jaitin, D. A. *et al*. Massively parallel single-cell RNA-seq for marker-free decomposition of tissues into cell types. *Science* **343**, 776–779, 10.1126/science.1247651 (2014). 24531970 10.1126/science.1247651 PMC4412462

[20] Lavin, Y. *et al*. Innate Immune Landscape in Early Lung Adenocarcinoma by Paired Single-Cell Analyses. *Cell* **169**, 750–765.e717, 10.1016/j.cell.2017.04.014 (2017). 28475900 10.1016/j.cell.2017.04.014 PMC5737939

[21] Dobin, A. *et al*. STAR: ultrafast universal RNA-seq aligner. *Bioinformatics* **29**, 15–21, 10.1093/bioinformatics/bts635 (2013). 23104886 10.1093/bioinformatics/bts635 PMC3530905

[22] R Core Team. R: A Language and Environment for Statistical Computing (R Foundation for Statistical Computing, Vienna, Austria, 2021).

[23] Liao, Y., Smyth, G. K. & Shi, W. The R package Rsubread is easier, faster, cheaper and better for alignment and quantification of RNA sequencing reads. *Nucleic Acids Res* **47**, e47, 10.1093/nar/gkz114 (2019). 30783653 10.1093/nar/gkz114 PMC6486549

[24] Love, M. I., Huber, W. & Anders, S. Moderated estimation of fold change and dispersion for RNA-seq data with DESeq. 2. *Genome Biol* **15**, 550, 10.1186/s13059-014-0550-8 (2014). 25516281 10.1186/s13059-014-0550-8 PMC4302049

[25] Hanzelmann, S., Castelo, R. & Guinney, J. GSVA: gene set variation analysis for microarray and RNA-seq data. *BMC Bioinformatics* **14**, 7, 10.1186/1471-2105-14-7 (2013). 23323831 10.1186/1471-2105-14-7 PMC3618321

[26] Hafemeister, C. & Satija, R. Normalization and variance stabilization of single-cell RNA-seq data using regularized negative binomial regression. *Genome Biol* **20**, 296, 10.1186/s13059-019-1874-1 (2019). 31870423 10.1186/s13059-019-1874-1 PMC6927181

[27] Stuart, T. *et al*. Comprehensive Integration of Single-Cell Data. *Cell* **177**, 1888–1902 e1821, 10.1016/j.cell.2019.05.031 (2019). 31178118 10.1016/j.cell.2019.05.031 PMC6687398

[28] Becht, E. *et al*. Dimensionality reduction for visualizing single-cell data using UMAP. *Nat Biotechnol*10.1038/nbt.4314 (2018). 30531897 10.1038/nbt.4314

[29] Aibar, S. *et al*. SCENIC: single-cell regulatory network inference and clustering. *Nat Methods* **14**, 1083–1086, 10.1038/nmeth.4463 (2017). 28991892 10.1038/nmeth.4463 PMC5937676

[30] Finak, G. *et al*. MAST: a flexible statistical framework for assessing transcriptional changes and characterizing heterogeneity in single-cell RNA sequencing data. *Genome Biol* **16**, 278, 10.1186/s13059-015-0844-5 (2015). 26653891 10.1186/s13059-015-0844-5 PMC4676162

[31] La Manno, G. *et al*. RNA velocity of single cells. *Nature* **560**, 494–498, 10.1038/s41586-018-0414-6 (2018). 30089906 10.1038/s41586-018-0414-6 PMC6130801

[32] Bergen, V., Lange, M., Peidli, S., Wolf, F. A. & Theis, F. J. Generalizing RNA velocity to transient cell states through dynamical modeling. *Nat Biotechnol* **38**, 1408–1414, 10.1038/s41587-020-0591-3 (2020). 32747759 10.1038/s41587-020-0591-3

[33] Calcagno, D. M. *et al*. Single-cell and spatial transcriptomics of the infarcted heart define the dynamic onset of the border zone in response to mechanical destabilization. *Nature Cardiovascular Research* **1**, 1039–1055, 10.1038/s44161-022-00160-3 (2022). 39086770 10.1038/s44161-022-00160-3 PMC11290420

[34] *NCBI Gene Expression Omnibus* https://identifiers.org/geo/GSE261428 (2025).

[35] *NCBI Gene Expression Omnibus* https://identifiers.org/geo/GSE267256 (2025).

[36] *NCBI Gene Expression Omnibus* https://identifiers.org/geo/GSE265828 (2025).

[37] Huang, C. *et al*. Asporin, an extracellular matrix protein, is a beneficial regulator of cardiac remodeling. *Matrix Biol* **110**, 40–59, 10.1016/j.matbio.2022.04.005 (2022). 35470068 10.1016/j.matbio.2022.04.005 PMC10234622

